# Estimating the Burden of Child Undernutrition for Smaller Electoral Units in India

**DOI:** 10.1001/jamanetworkopen.2021.29416

**Published:** 2021-10-29

**Authors:** Julie Kim, Yuning Liu, Weiyu Wang, Jeffrey C. Blossom, Laxmi Kant Dwivedi, K. S. James, Rakesh Sarwal, Rockli Kim, S.V. Subramanian

**Affiliations:** 1 Harvard Center for Population and Development Studies, Harvard University, Cambridge, Massachusetts; 2 JP Morgan Chase Institute, Washington, DC; 3 Center for Geographic Analysis, Harvard University, Cambridge, Massachusetts; 4 International Institute for Population Sciences, Deonar, Mumbai, Maharashtra, India; 5 National Institution for Transforming India Aayog. Government of India, New Delhi, India; 6 Division of Health Policy and Management, College of Health Science, Korea University, Seoul, South Korea; 7 Interdisciplinary Program in Precision Public Health, Department of Public Health Sciences, Graduate School of Korea University, Seoul, South Korea; 8 Department of Social and Behavioral Sciences, Harvard T.H. Chan School of Public Health, Boston, Massachusetts

## Abstract

**Importance:**

Geographic targeting of public health interventions is needed in resource-constrained developing countries.

**Objective:**

To develop methods for estimating health and development indicators across micropolicy units, using assembly constituencies (ACs) in India as an example.

**Design, Setting, and Participants:**

This cross-sectional study included children younger than 5 years who participated in the fourth National Family and Health Survey (NFHS-4), conducted between January 2015 and December 2016. Participants lived in 36 states and union territories and 640 districts in India. Children who had valid weight and height measures were selected for stunting, underweight, and wasting analysis, and children between age 6 and 59 months with valid blood hemoglobin concentration levels were included in the anemia analysis sample. The analysis was performed between February 1 and August 15, 2020.

**Exposures:**

A total of 3940 ACs were identified from the geographic location of primary sampling units in which the children’s households were surveyed in NFHS-4.

**Main Outcomes and Measures:**

Stunting, underweight, and wasting were defined according to the World Health Organization Child Growth Standards. Anemia was defined as blood hemoglobin concentration less than 11.0 g/dL.

**Results:**

The main analytic sample included 222 172 children (mean [SD] age, 30.03 [17.01] months; 114 902 [51.72%] boys) from 3940 ACs in the stunting, underweight, and wasting analysis and 215 593 children (mean [SD] age, 32.63 [15.47] months; 112 259 [52.07%] boys) from 3941 ACs in the anemia analysis. The burden of child undernutrition varied substantially across ACs: from 18.02% to 60.94% for stunting, with a median (IQR) of 35.56% (29.82%-42.42%); from 10.40% to 63.24% for underweight, with a median (IQR) of 32.82% (25.50%-40.96%); from 5.56% to 39.91% for wasting, with a median (IQR) of 19.91% (15.70%-24.27%); and from 18.63% to 83.05% for anemia, with a median (IQR) of 55.74% (48.41%-63.01%). The degree of inequality within states varied across states; those with high stunting, underweight, and wasting prevalence tended to have high levels of inequality. For example, Uttar Pradesh, Jharkhand, and Karnataka had high mean AC-level prevalence of child stunting (Uttar Pradesh, 45.29%; Jharkhand, 43.76%; Karnataka, 39.77%) and also large SDs (Uttar Pradesh, 6.90; Jharkhand, 6.02; Karnataka, 6.72). The Moran *I* indices ranged from 0.25 to 0.80, indicating varying levels of spatial autocorrelation in child undernutrition across the states in India. No substantial difference in AC-level child undernutrition prevalence was found after adjusting for possible random displacement of geographic location data.

**Conclusions and Relevance:**

In this cross-sectional study, substantial inequality in child undernutrition was found across ACs in India, suggesting the importance of considering local electoral units in designing targeted interventions. The methods presented in this paper can be further applied to measuring health and development indicators in small electoral units for enhanced geographic precision of public health data in developing countries.

## Key Points

**Question:**

How can health and development indicators be estimated across micropolicy units when geographic identifiers are not available in nationally representative surveys?

**Findings:**

In this cross-sectional study of 222 172 children in 3940 assembly constituencies (ACs) in India, a high degree of inequality in child undernutrition was found across ACs, with a substantial variation within states and dissimilar patterns of geographic clustering.

**Meaning:**

These findings suggest that local electoral units should be considered when designing geographically targeted interventions to reduce the burden of child undernutrition.

## Teaser

This cross-sectional study estimates health and development indicators across micropolicy units using a newly developed method.

## Introduction

Child undernutrition, a major contributor to deaths among children younger than 5 years in developing countries, has dire effects that extend beyond the realm of public health.^1,2^ The causes of child undernutrition are diverse and ultimately rooted in socioeconomic determinants that are unequally distributed across and within countries.^3,4^ To make meaningful progress in global child undernutrition targets and related Sustainable Development Goals, public health data should be mobilized in a way that enhances geographic precision.^5,6,7^ Where resources are constrained, estimates of child undernutrition at a very fine scale can enable identification of high burden areas and guide decisions on resource allocation.^8,9^

At the same time, the political and administrative functionality of a given geographical unit should be considered to maximize the practical utility of data.^10^ Monitoring health and development indicators at local electoral units has the potential to facilitate evidence-based decision-making and to improve governmental accountability. In many developing countries, lack of political commitment has been recognized as a primary reason for inadequate investment on public health and nutrition policies.^11,12,13,14^ Provision of a monitoring framework that constituents can use to assess political representatives’ performance and responsiveness is a promising way to promote greater responsibility among decision-makers.

In most countries, however, data availability has pivoted development monitoring and policy implementation to the macro level, ie, administrative unit boundaries, such as states or provinces. For example, Demographic and Health Surveys (DHS), the Census, and Living Standards and Measurement Surveys (LSMS) all include administrative unit identifiers, while none of the regional identifiers are related to political units.^15,16,17^

In India, where more than 35% of children are estimated to have stunted growth and nearly 70% of deaths among children younger than 5 years are attributed to inadequate nutrition,^18^ there has been a growing interest in developing methods to make health data available at the electoral unit of the parliamentary constituencies (PCs).^19,20,21^ However, PCs are fairly large units, with substantial heterogeneity within them.^22^ Assembly constituencies (ACs) are local electoral units that remain unexplored. Directly elected from each AC, the members of the Legislative Assembly (MLAs) constitute the legislature of states or union territories (UTs). Although the scope of their authority is not clearly laid out in the constitution, MLAs often raise issues of public importance, participate in legislative business in the state assembly, vote on demands for grants in the budget, and decide on governance lapses, as brought by audit reports by the comptroller and auditor general. Hence, ACs are important units at which mobilization of data can potentially enhance geographic precision and the political accountability of local authorities.

This article introduces a method to estimate health and development metrics at local electoral units using ACs in India as a geographical unit of measurement. Multilevel modeling was used to produce precision-weighted prevalence rates of stunting, underweight, wasting, and anemia at the AC level using the fourth round of National Family and Health Survey (NFHS-4) data. The distribution of these estimates within larger levels of geography are summarized. The monitoring framework presented in this study can be applied to other indicators of interest to enhance political commitment with greater geographic precision.

## Methods

This study was reviewed by the Harvard T.H. Chan School of Public Health institutional review board and was considered exempt from full review and the requirement for informed consent, as the study used an anonymous public data set with no identifiable information on the study participants. The analysis was performed between February 1 and August 15, 2020, following the Strengthening the Reporting of Observational Studies in Epidemiology (STROBE) reporting guideline.

### Data Source

**Figure 1.**
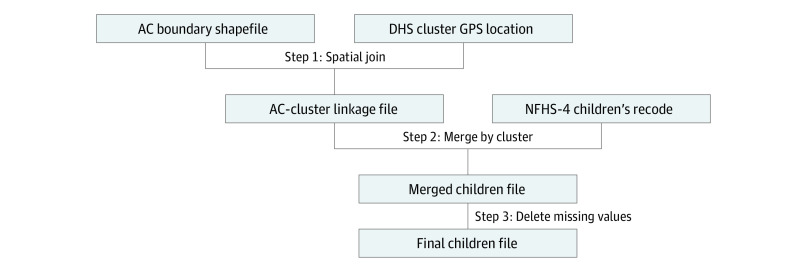
Flowchart of the Process of Assembly Constituency (AC)–Level Estimation DHS indicates Demographic and Health Surveys; GPS, global positioning system; NFHS-4, fourth National Family and Health Survey.

Three data sources were used to create the final analytic sample (Figure 1). The first data source was NFHS-4 for data on undernutrition among children. As part of the global DHS framework, India’s NFHS-4 is a nationally representative survey that contains records of 259 267 children from 601 509 households, with information on their sociodemographic characteristics, risk factors, health service utilization, and health outcomes; the NFHS-4 was conducted from 2015 to 2016.^23^ The second data source was the geographic location data of the primary sampling units (ie, clusters) accessed via special request from DHS. The last data source was the boundary shapefiles for ACs and states, obtained from Datameet project published in Github.^24^ A total of 4148 AC polygons were identified, among which 33 were marked as null and 1 polygon contained 5 ACs. These unidentifiable ACs were dropped.

### Child Undernutrition Indicators

Four indicators were considered to estimate the burden of child malnutrition in India^1^: children younger than 5 years with stunting, defined as height-for-age less than −2 SDs of the age- and sex-specific median according to the World Health Organization (WHO) Child Growth Standards^2^; children younger than 5 years with underweight, defined as weight-for-age below −2 SD of the age- and sex-specific median following the same WHO standard^3^; children younger than 5 years with wasting, defined as weight-for-height below −2 SD of the age- and sex-specific median^4^; and children aged 6 to 59 months who had anemia, defined as having hemoglobin concentration less than 11.0 g/dL (to convert to grams per liter, multiply by 10.0).^25^ These indicators were selected because the government of India is currently monitoring them as part of the National Nutrition Mission (NNM or the *POSHAN Abhiyaan*) with specific targets of reducing child undernutrition by at least 2% per annum.^26^ We used R version 3.6.2 (R Project for Statistical Computing) for this computation.

### Linking NFHS-4 Data to AC

To assign AC membership of NFHS-4 clusters, the first step was to conduct spatial join between NFHS-4 clusters and ACs boundary shapefile, as developed in a previous research focused on PCs (Figure 1).^20^ The NFHS-4 cluster membership to AC was defined as the cluster geographically nested within the AC boundary. A second set of cluster AC memberships was established, adjusting for the random displacement policies of DHS data. This sample is referred to as the reassignment sample. For privacy protection, DHS conducted random displacement by as much as 2 km for urban and 5 km for rural clusters.^27,28^ A buffer with a radius of displacement was created, in which the buffers were intersected by superimposed AC boundaries. The ratio between the area of segments in different ACs was calculated, and AC membership was assigned to the AC with the highest ratio. In our data, 15 013 of 259 627 children (5.97%) were reassigned, indicating a minor discrepancy between the nonadjustment and reassignment sample. Overall, 28 059 of 28 256 clusters (98.36%) and 27 989 of 28 256 clusters (98.11%) remained after the spatial join process for nonadjustment and reassignment samples, respectively. Less than 2% of clusters were dropped in this process; clusters were dropped for the following reasons: missing longitude or latitude in the DHS geographic file, falling outside of the AC boundary shapefile, or falling in an AC not identified in the source shapefile (ie, no AC name present). This linkage was performed in ArcGIS Pro version 2.0 (Esri).

The next step was to link the NFHS-4 data file to the merged data set in which DHS cluster membership to AC was identified. Overall, 27 872 clusters (97.71%) and 27 802 clusters (97.46%) in the nonadjustment and reassignment samples, respectively, remained after this process, corresponding to 256 353 of 259 627 children (98.74%) and 255 744 of 259 627 children (98.50%) in those samples, respectively. The minor exclusion of clusters and children occurred for some clusters that had no children in the sample and when AC-cluster linkage information was not identified in the cluster in which the children were sampled.

For the last step of data processing, children who did not have information on age, height, weight, or blood hemoglobin levels were excluded. After removing missing observation for height and weight, 221 172 children (85.57%) and 221 622 children (85.36%) remained in the nonadjustment and reassignment samples, respectively. After removing entries without blood hemoglobin measurement, 215 593 children (83.04%) and 215 075 children (82.84%) remained in the nonadjustment and reassignment samples, respectively. The Table shows the final number of children, clusters, ACs, and states/UTs.

**Table.** Number of Samples at Each Step of AC-Level Estimation

Step	No. (%)			
	Children (N = 259 627)	Cluster (N = 28 256)	AC (N = 4120)	State/UT (N = 36)
**Nonadjustment**				
Step 1	NA	28 059 (98.36)	3959 (94.62)	32 (88.89)
Step 2	256 353 (98.74)	27 872 (97.71)	3950 (95.19)	32 (88.89)
Step 3				
Anemia analytic sample	215 593 (83.04)	27 743 (96.97)	3941 (96.02)	32 (88.89)
Stunting, underweight, and wasting analytic sample	222 172 (85.57)	27 711 (97.25)	3940 (95.63)	32 (88.89)
**Reassignment**				
Step 1	NA	27 989 (98.11)	3909 (94.88)	31 (86.11)
Step 2	255 744 (98.50)	27 802 (97.46)	3901 (93.90)	31 (94.44)
Step 3				
Anemia analytic sample	215 075 (82.84)	27 674 (97.43)	3894 (93.81)	31 (94.44)
Stunting, underweight, and wasting analytic sample	221 622 (85.36)	27 642 (97.70)	3894 (93.86)	31 (94.44)

Abbreviations: AC, assembly constituencies; NA, not applicable; UT, union territory.

### Statistical Analysis

AC-level prevalence of stunting, underweight, wasting, and anemia was calculated using a random-effects model, also known as a multilevel model, that fully accounts for the sampling design of NFHS-4. More specifically, a 4-level model was constructed with children (level 1) nested within NFHS-4 clusters (level 2), within districts (level 3), and within states (level 4). Using this model, cluster-specific estimates were computed using Monte Carlo Markov chain method and Gibbs sampler with noninformative priors, a burn-in of 5000 cycles, and monitoring of 50 000 iterations of chains.^29^ The mean of cluster-specific estimates across the clusters that share the same membership to ACs was computed. More details on the precision-weighted estimation are presented in eAppendix 1 in Supplement 1. All multilevel modeling was performed using MLwiN version 3.05 (Centre for Multilevel Modeling, University of Bristol).

The variation of AC-level child undernutrition across different geographical units was summarized in various ways. First, the IQR of AC-level undernutrition prevalence was estimated. Second, the level of geographical clustering was measured by global Moran *I* index, using the queen contiguity weights in each state and across all India. Statistical significance for Moran *I* statistics was calculated from a 2-tailed *P *value, with *P* < .05 as the level of statistical significance. Finally, the SD of AC-level prevalence was calculated for each state. All three analyses were conducted using R version 3.6.2 (R Project for Statistical Computing).

To check for robustness of our models, additional analysis was performed using a 5-level hierarchical model: child (level 1), cluster (level 2), AC (level 3), district (level 4), and state (level 5). Because ACs do not hierarchically nest within districts, AC-district membership was adjusted using the following criteria. If an AC was nested within multiple districts, the district that covered more than 60% of clusters was chosen. If an AC spanned multiple districts and the number of clusters was spread relatively equally, the district that covered the largest geographical area was chosen. AC-level prevalence of child undernutrition calculated from this 5-level model was compared with our main analysis, which used 4-level model without AC random effects.

## Results

There were 222 172 children (mean [SD] age, 30.03 [17.01] months; 114 902 [51.72%] boys) in the stunting, underweight, and wasting analysis, corresponding to 3940 ACs. There were 215 593 children (mean [SD] age, 32.63 [15.47] months; 112 259 [52.07%] boys) in the anemia analysis, corresponding to 3941 ACs. The children lived in 32 states/UTs.

### Distribution of Undernutrition Indicators in India

**Figure 2.**
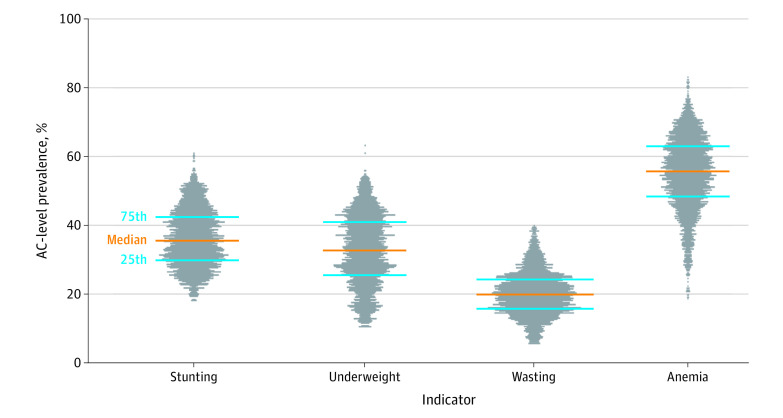
Distribution of Child Undernutrition Prevalence in Assembly Constituencies (ACs) in 2016

Figure 2 shows the distribution of AC-level prevalence of 4 child undernutrition indicators. AC-level prevalence of child stunting ranged from 18.02% to 60.94%, with a median prevalence of 35.56% and an IQR from 29.82% to 42.42%; child underweight ranged from 10.40% to 63.24%, with a median of 32.82% and an IQR from 25.50% to 40.96%; wasting ranged from 5.56% to 39.91%, with a median of 19.91% and an IQR from 15.70% to 24.27%; lastly, child anemia ranged from 18.63% to 83.05%, with a median of 55.74% and an IQR from 48.41% to 63.01%. The estimates from reassignment sample were very similar to those from non-adjustment sample (Supplement 2). We also provide an interactive view of the AC maps in a dashboard where users can view the prevalence of child undernutrition for a selected AC.^30^

### Distribution of Child Undernutrition Indicators Across ACs

**Figure 3.**
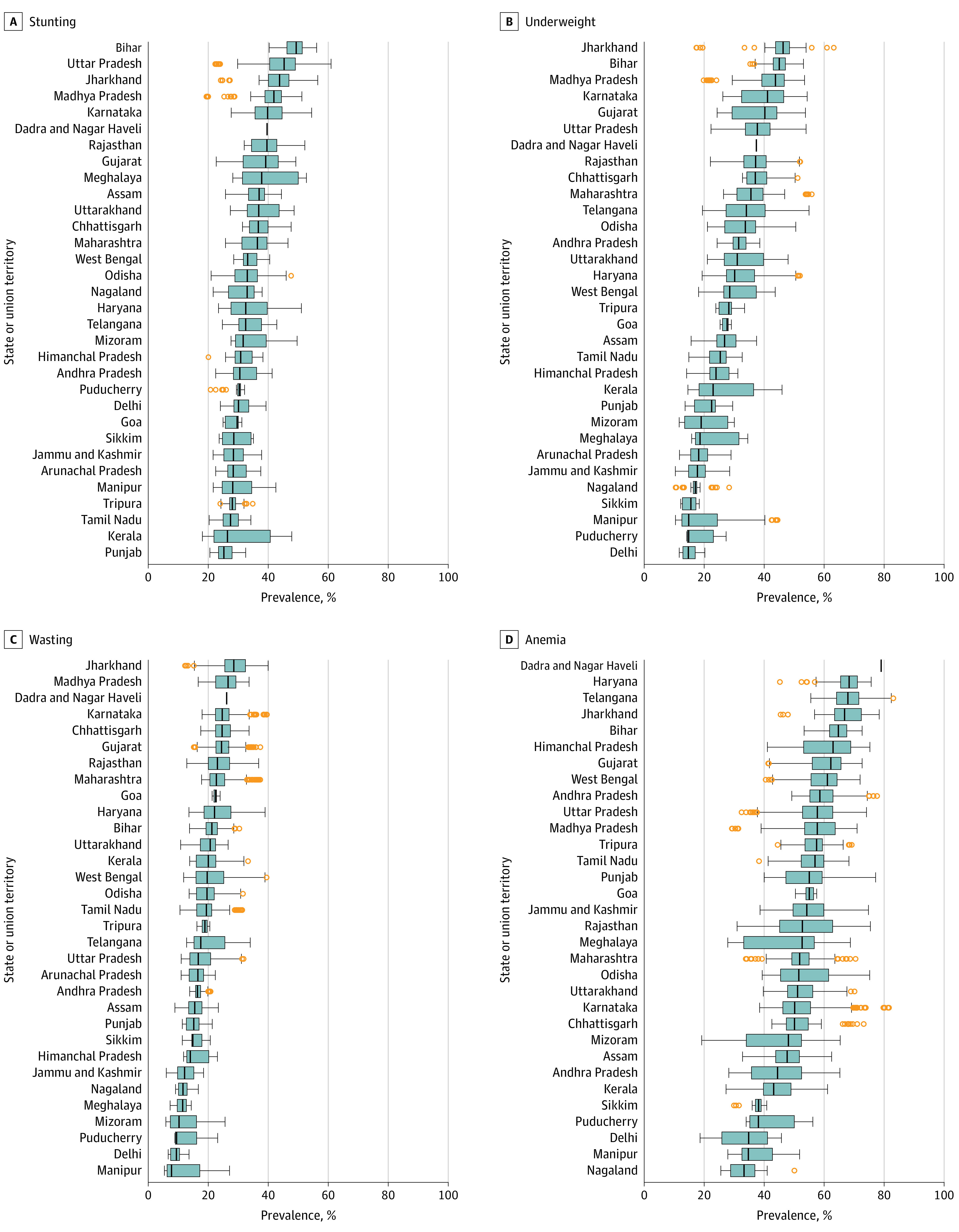
Distribution of Assembly Constituency–Level Prevalence for Stunting, Underweight, Wasting, and Anemia Within Each State/Union Territory in 2016 The boxplot represents the 25th, 50th, and 75th percentiles of assembly constituency–level prevalence within each state/union territory. The orange dots indicate outliers. The whiskers indicate the range. If there are no outliers, the left end of the whisker indicates the minimum value and the right end of the whisker the maximum. If there is an outlier, the left end indicates the 25th percentile value minus 1.5 times the IQR and the right end indicates the 75th percentile plus 1.5 times the IQR.

Figure 3 shows the medians and IQRs of AC-level prevalence rates within each state/UT. Considering the median of AC-level prevalence, Jharkhand ranked the highest for underweight (46.34%) and wasting (28.55%), the third highest for stunting (43.76%), and the fourth highest in anemia (66.82%). Bihar ranked the highest in stunting (49.32%) and the second highest for underweight (45.07%), and Madhya Pradesh ranked the second highest in wasting (26.65%), the third highest in underweight (43.79%), and the fourth highest in stunting (41.87%). Meanwhile, Delhi, Puducherry, and Manipur tended to have low levels of state medians.

The magnitude of spread in the AC-level prevalence within states/UTs varied (Figure 3). For instance, Bihar had higher median stunting prevalence (49.32%) than Uttar Pradesh (44.08%), but its IQR (46.23%-51.32%) was much smaller than that of Uttar Pradesh (40.45%-49.03%). Similarly, Manipur had the lowest median wasting prevalence, but its IQR and range both exceeded those of several states that had higher median wasting prevalence, including Delhi, Puducherry, Punjab, and Assam.

Stunting, underweight, and wasting showed similar patterns in the association between the mean AC-level prevalence and its SD, while anemia showed a different pattern (eAppendix 2 in Supplement 1). Jharkhand, Karnataka, and Madhya Pradesh had high mean AC-level prevalence of stunting, underweight, and wasting (eg, stunting: Uttar Pradesh, 45.29%; Jharkhand, 43.76%; Karnataka, 39.77%) and also larger SDs (eg, stunting: Uttar Pradesh, 6.90; Jharkhand, 6.02; Karnataka, 6.72), indicating that the inequality across AC-level burden was greater in the states with larger burden. However, anemia did not show the same pattern. States/UTs with a high prevalence of anemia, including Haryana, Bihar, and Telangana, all had relatively smaller SDs, while lower-burden states, such as Meghalaya or Mizoram, had relatively higher SDs.

### Geographical Patterns of Undernutrition Indicators

**Figure 4.**
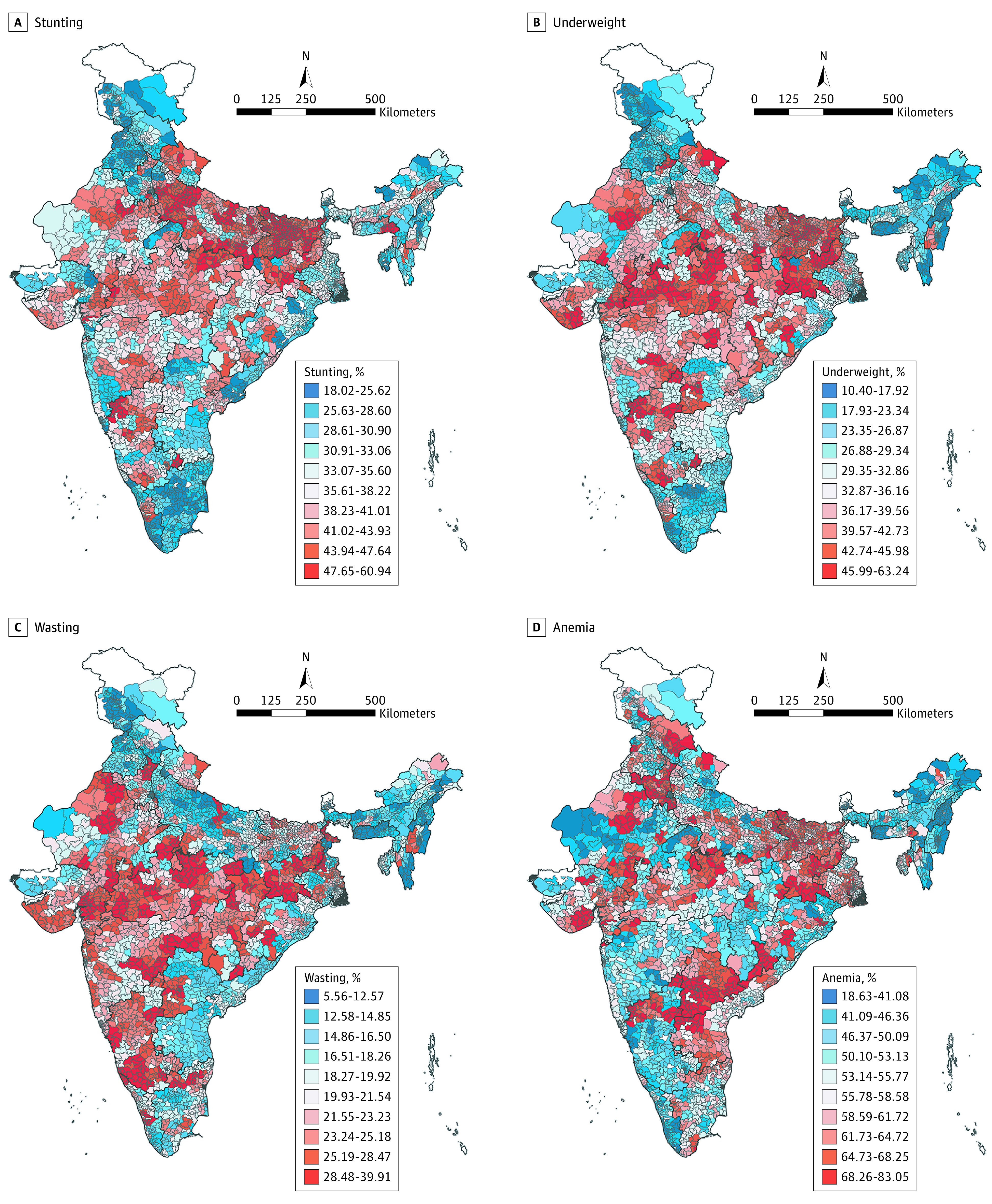
Precision-Weighted Prevalence of Child Undernutrition Across Assembly Constituencies in India Assembly constituency–level prevalence was classified into deciles (10 classes), from the lowest (blue) to the highest (red).

Figure 4 shows geographical distribution of AC-level prevalence of child stunting (Figure 4A), underweight (Figure 4B), wasting (Figure 4C), and anemia (Figure 4D) across India. ACs in Southern India had relatively low prevalence of all 4 indicators, while the burden was highly concentrated in central and western India. Stunting, underweight, and wasting showed similar geographical distribution, such that the burden was highly concentrated in the western and southern parts of central India. While ACs in northern India had lower deciles of stunting, underweight, and wasting, they had notably higher prevalence of anemia.

Results from spatial autocorrelation analysis showed varying degrees of geographical clustering of child undernutrition burden across the states in India (eAppendix 3 in Supplement 1). High Moran *I* index across all 4 indicators at the national level (all India) indicated a high level of clustering overall. Meghalaya, Kerala, Tripura, and Gujarat had high Moran *I* indices for all 4 malnutrition indicators (stunting: Meghalaya, 0.87; Kerala, 0.84; Tripura, 0.81; and Gujarat, 0.79). Among the 4 indicators, anemia tended to have lower Moran *I* values, indicating a relatively lower degree of geographical clustering. The *P* value of Moran *I* index for all indicators across every state/UT, including all of India, was significant (*P* <.001), except for stunting in Puducherry (Moran *I*, 0.096), which was *P* = .11.

### Sensitivity Analysis

Comparison between a 4-level model and a 5-level model with AC random effects revealed that the 2 models produced similar AC-level prevalence of child undernutrition. The mean (SD) difference in prevalence resulting from the 2 models was 3.02 (2.71), 3.09 (2.68), 2.53 (2.25), and 4.44 (4.08) percentage points for stunting, underweight, wasting, and anemia, respectively, indicating that the precision-weighted AC-level estimates from the 5-level model did not substantially differ from the 4-level model used for our main analysis.

## Discussion

The primary objective of this article was to demonstrate a method that can be used to estimate health and development indicators at important small electoral units within India. Three salient findings emerged from our analysis of AC-level burden of child undernutrition in India. First, the prevalence of child undernutrition varied substantially across ACs, reaching differences of more than 50 percentage points between ACs with the highest and the lowest rates. Second, states in central and western India, in general, had the highest burden of child undernutrition. Third, levels of inequality and the extent of geographic clustering varied across states. That is, some states with low state-level burden had a high level of variation, while some states with low state-level burden had a relatively homogeneous distribution within them. All of these findings suggest the importance of improving geographic precision in public health data in developing countries.

Upon visual inspection, the overall distribution of ACs with high burdens was comparable to a prior geospatial analysis of child undernutrition estimates by 5 × 5 km grids.^9^ One way to explain local heterogeneity of child undernutrition is the varying degrees of access to resources. Previous epidemiologic studies have consistently found a strong association between socioeconomic status and child undernutrition outcomes in many developing countries,^31,32,33^ including India.^4,34^ Indeed, states with the highest burden of child undernutrition identified in our study were also those with the lowest per capita GDP (ie, Bihar, Uttar Pradesh, and Jharkhand). The extent to which the observed geographic variability in AC-level child undernutrition can be explained by established risk factors was beyond the scope of the present study, but it should be investigated in future studies.

To our knowledge, this is the first method developed for AC-level estimation of health and development indicators. While federal policies provide directionality and frameworks, state legislatures in India have exclusive powers to initiate and reinforce laws in various domains, including public health, sanitation, and agriculture. Therefore, data at the AC level and distribution across ACs within each state can help local policy makers better represent their population and design policies according to local needs. For example, the wide variation in AC-level child undernutrition within certain states suggests that each state faces different types of challenge. Even for states with on-average low burden, the large degree of inequality indicates that different local targeting strategies are needed to reduce the burden of child undernutrition. This kind of tailored approach can help India achieve its target of reducing child undernutrition by at least 2% per annum, as specified in the NNM.

Another important contribution of this study is the provision of a monitoring framework that can improve governmental accountability. Monitoring at the local electoral unit can help policy makers better understand the public health needs of the population they represent and simultaneously help voters evaluate progress. A monitoring framework in which political leaders can be held accountable could be the key to increasing political engagement.

### Limitations

The proposed method and the resulting AC estimates should be interpreted with the following limitations. The possibility of incorrect linkage between NFHS-4 clusters and AC boundaries remains an important limitation. This is inevitable given the random displacement of NFHS-4 cluster points. To address such uncertainty, additional analysis was performed accounting for the probability of misplacement. Consistent results were found between nonadjustment and reassignment samples, suggesting that the estimates presented in this study are robust. However, it is not possible to fully address the uncertainty caused by the random displacement. It is also important to note that the NFHS-4 data were collected over a 2-year period (2015-2016), and the burden of undernutrition can be highly influenced by seasonal variation. The differential timing of data collection is more likely to affect acute malnutrition (wasting) than chronic malnutrition (stunting).

## Conclusions

In this study, the large local variation in child undernutrition burden in India suggests the need for policy design, monitoring, and evaluation at smaller geographic units with political and electoral functions. With enhanced geographic precision, the data presented in this study visualize the ACs that must be prioritized to effectively reduce the burden of child undernutrition in India.

## Notes

Supplement 1. **eAppendix 1. **Methodological Note on Using Multilevel Modeling for Precision-Weighted Estimates **eAppendix 2. **Mean and SD of AC-Level Prevalence Across States in India **eAppendix 3. **Spatial Autocorrelation of Child Malnutrition Indicators by State

Supplement 2.
Child Undernutrition Prevalence Across Assembly Constituencies in India
