# Supplementary material for: Estimating the Burden of Child Undernutrition for Smaller Electoral Units in India
Source: JAMA Netw Open. 2021 Oct 29;4(10):e2129416. doi: 10.1001/jamanetworkopen.2021.29416 (PMC8556624; doi:10.1001/jamanetworkopen.2021.29416)
Supplement: Supplement 1. — eAppendix 1. Methodological Note on Using Multilevel Modeling for Precision-Weighted Estimates eAppendix 2. Mean and SD of AC-Level Prevalence Across States in India eAppendix 3. Spatial Autocorrelation of Child Malnutrition Indicators by State [file jamanetwopen-e2129416-s001.pdf]

Supplemental Online Content

Kim J, Liu Y, Wang W, et al. Estimating the burden of child undernutrition for smaller electoral units in India. *JAMA Netw Open.* 2021;4(10):e2129416.
doi:10.1001/jamanetworkopen.2021.29416

eAppendix 1. Methodological Note on Using Multilevel Modeling for Precision-Weighted Estimates

eAppendix 2. Mean and SD of AC-Level Prevalence Across States in India

eAppendix 3. Spatial Autocorrelation of Child Malnutrition Indicators by State

This supplemental material has been provided by the authors to give readers additional information about their work.

© 2021 Kim J et al. *JAMA Network Open.*

eAppendix 1. Methodological Note on Using Multilevel Modeling for Precision-Weighted Estimates

Precision-weighted estimates of child stunting, wasting, underweight, and anemia were generated based on the four-level random intercept logistic model:

$$logit(\pi_{ijkl}) = \beta + (u_{jkl} + v_{kl} + f_l)$$

Where

- π_{ijkl} denotes the probability of outcome Y for a child i (level-1) in cluster j (level-2) in district k (level-3), and state l (level-4)
- β denotes the overall mean logit probability of outcome Y
- u_{jkl} denotes cluster-specific residuals with a variance of σ_u^2 assuming $u_j \sim N(0, \sigma_u^2)$
- v_{kl} denotes district-specific residuals with a variance of σ_v^2 assuming $v_{kl} \sim N(0, \sigma_v^2)$
- f_l denotes state-specific residuals with a variance of σ_f^2 assuming $f_l \sim N\left(0, \sigma_f^2\right)$
- All the random effects are assumed to be independent of one another

Note: For binary outcome models, the variance at the individual level is approximated using a latent variable method as $\pi^2/3$.

From the model above, cluster-specific predicted logit values were converted to probabilities by taking the average over the simulations:

$$\exp\,(\beta_{jkl}) = \exp\big(\beta + (u_{jkl} + v_{kl} + f_l)\big)\,/(1 + \exp\big(\beta + (u_{jkl} + v_{kl} + f_l)\big)$$

Finally, the mean of cluster-specific predictions across the clusters that share the same membership to assembly constituencies (ACs) was computed for AC-level prevalence.

There are several advantages to this methodological approach. First, this four-level model accounts for the complex survey design and the resulting four-level hierarchical data structure. This model allows clusters from the same districts to be more similar to each other than they are to clusters from other districts. Second, all the information in the data are pooled to borrow strength such that poorly estimated cluster-specific predictions can benefit from the information for other clusters. That is, unreliable cluster-specific fixed estimates are differentially shrunken or smoothed towards the overall mean which is based on all the data and hence generate more appropriately conservative estimates.

© 2021 Kim J et al. *JAMA Network Open.*

eAppendix 2. Mean and SD of AC-Level Prevalence Across States in India

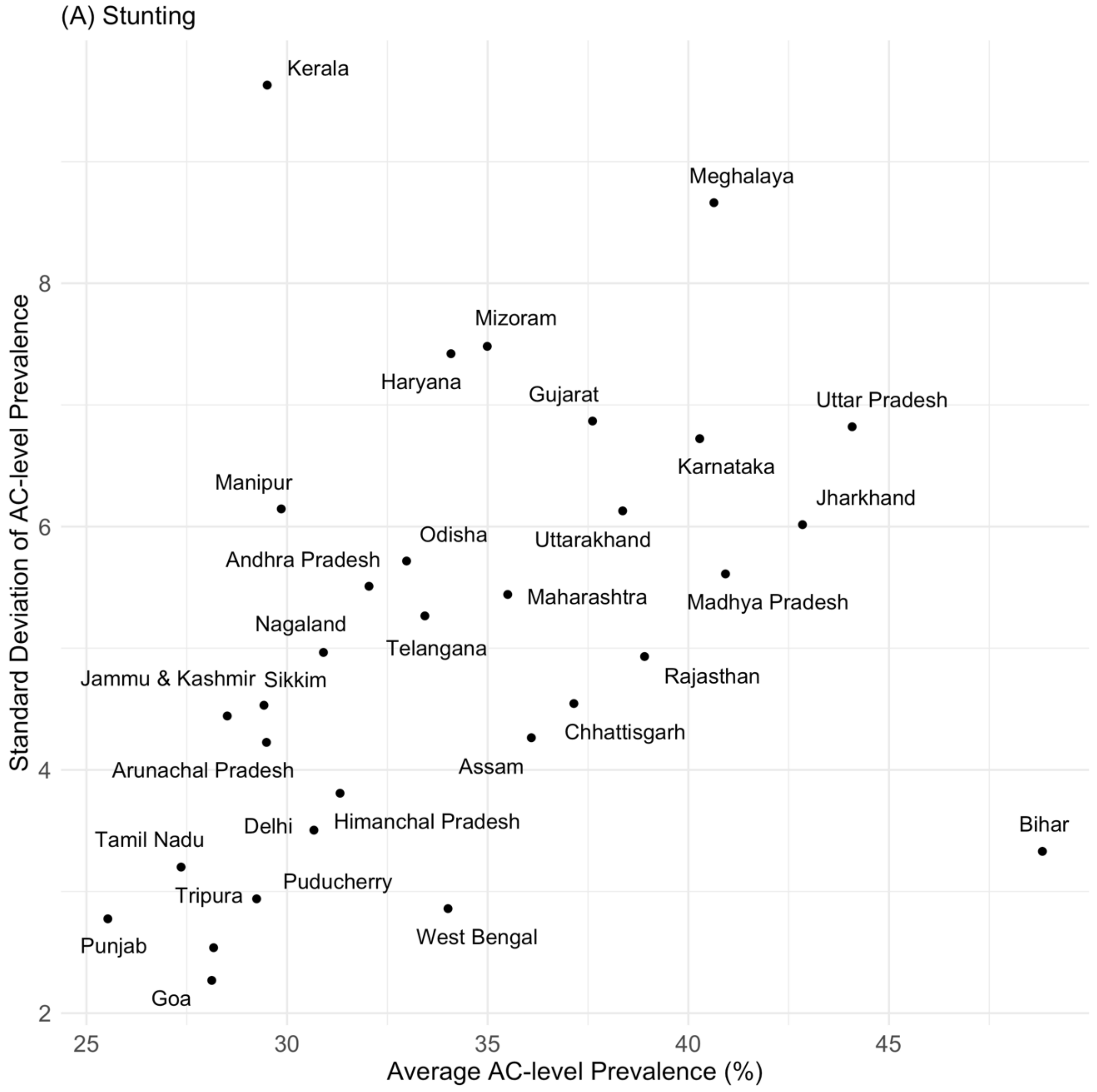

© 2021 Kim J et al. *JAMA Network Open.*

(B) Underweight

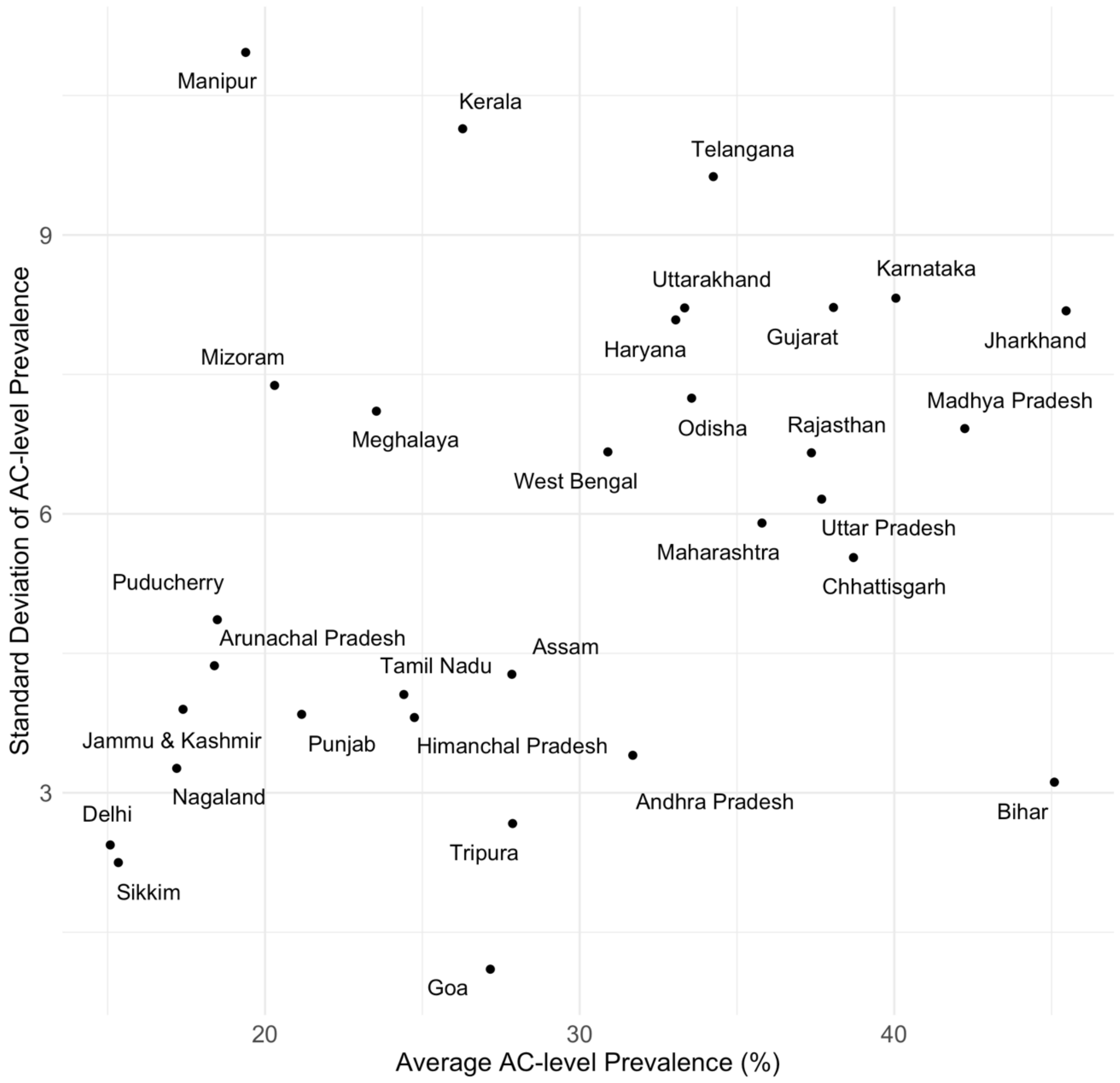

© 2021 Kim J et al. *JAMA Network Open.*

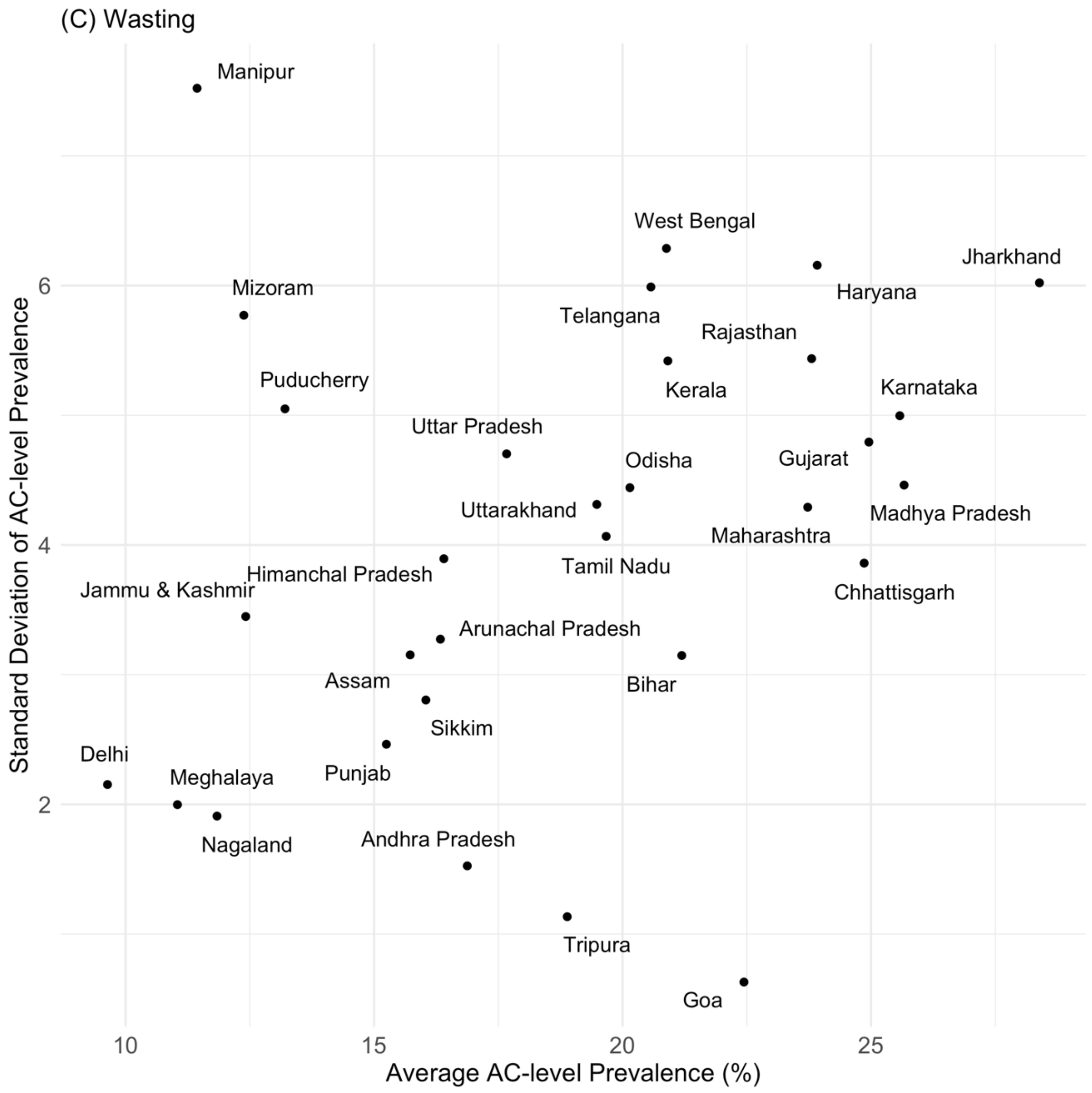

© 2021 Kim J et al. *JAMA Network Open.*

(D) Anemia

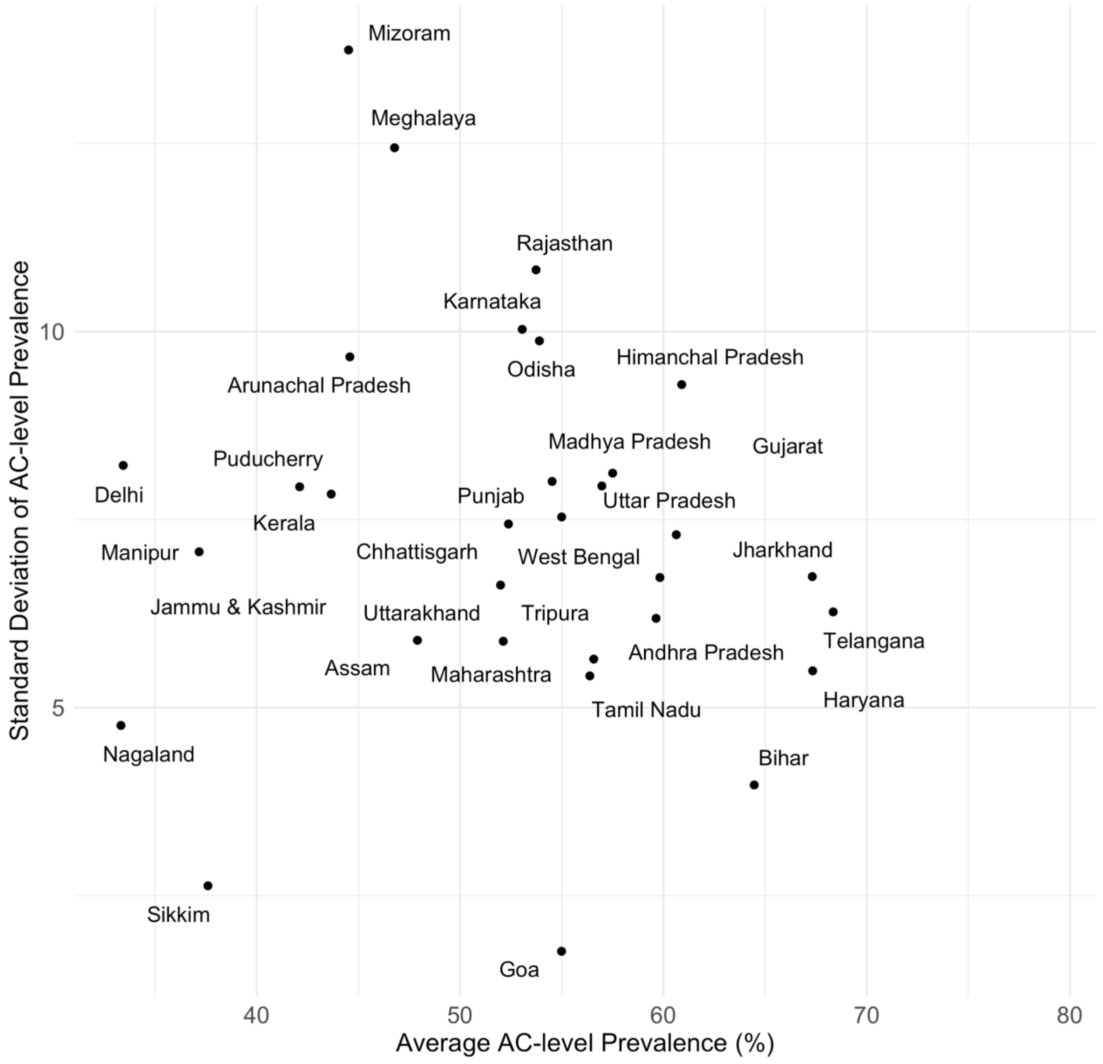

© 2021 Kim J et al. *JAMA Network Open.*

eAppendix 3. Spatial Autocorrelation of Child Malnutrition Indicators by State
Global Moran's I Indices for stunting, underweight, wasting, and anemia across all India (1st row) and in each state are presented.

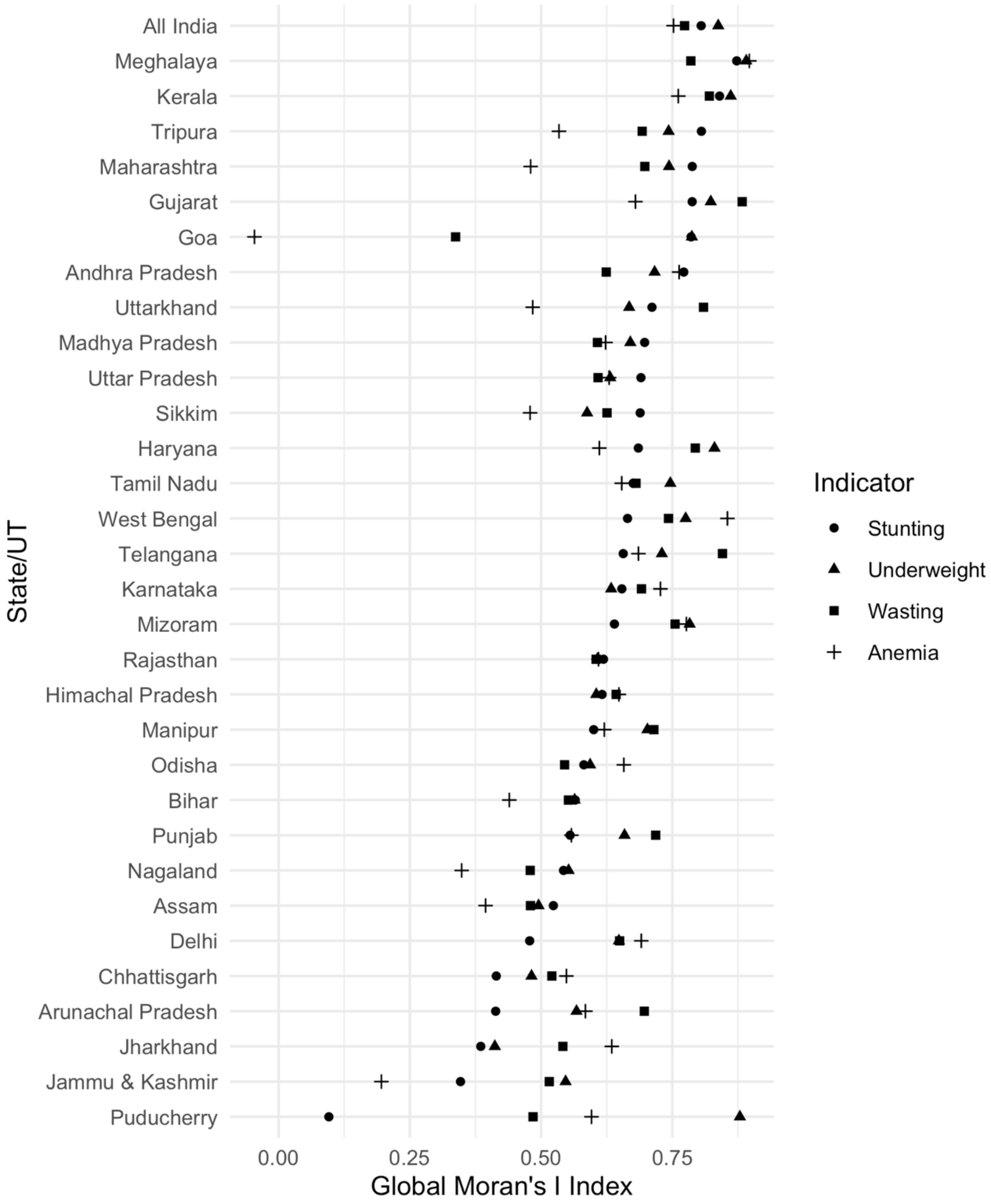

© 2021 Kim J et al. *JAMA Network Open.*
